# Association between Phase Angle and Subjective Perceptions of Health Variables in Cancer Patients

**DOI:** 10.3390/healthcare11131852

**Published:** 2023-06-26

**Authors:** Borja Gutiérrez-Santamaría, Aitor Martinez Aguirre-Betolaza, Arturo García-Álvarez, María Soledad Arietaleanizbeaskoa, Nere Mendizabal-Gallastegui, Gonzalo Grandes, Aitor Coca, Arkaitz Castañeda-Babarro

**Affiliations:** 1 Department of Physical Activity and Sport Sciences, Faculty of Education and Sport, University of Deusto, 48007 Bilbo, Bizkaia, Spain; borjagutierrez@deusto.es (B.G.-S.); a.martinezdeaguirre@deusto.es (A.M.A.-B.); arkaitz.castaneda@deusto.es (A.C.-B.); 2 Primary Care Research Unit of Bizkaia, Biocruces Bizkaia Health Research Institute, Plaza de Cruces 12, 48903 Barakaldo, Biscay, Spain; arturo.garciaalvarez@osakidetza.eus (A.G.-Á.); mariasoledad.arietaleanizbeaskoasarabia@osakidetza.eus (M.S.A.); nere.mandizabalgallastegui@osakidetza.eus (N.M.-G.); gonzalo.grandesodriozola@osakidetza.eus (G.G.); 3 Department of Physical Activity and Sports Sciences, Faculty of Health Sciences, Euneiz University, Vitoria-Gasteiz, Alava, La Biosfera Ibilbidea, 6, 01013 Gasteiz, Araba, Spain

**Keywords:** GHQ-12, FACIT, SF-36, bioimpedance, phase angle, early diagnosis, physical activity

## Abstract

The phase angle, an increasingly studied healthcare tool, was studied to explore its relationship with psychological factors in cancer patients. The aim of this study was to investigate the relationship between the phase angle (PhA), obtained by the bioimpedance analysis of body composition, and psychological factors measured by questionnaire in cancer patients. The study included 311 patients who underwent bioimpedance testing to determine their PhA value; their psychological profiles were assessed using SF-36, FACIT, QLQ-C30, and GHQ-12 questionnaires. Mixed linear regression models were used to analyze the relationship between PhA and the psychological tests. The results showed a statistical correlation between PhA and the GHQ-12, FACIT, and SF-36 questionnaires, with higher PhA values associated with better results on the questionnaires. In the QLQ-C30 questionnaire, a correlation was observed between PhA and the functioning scales (*p* < 0.001), except for emotional and cognitive functioning (*p* = 0.148 and *p* = 0.544, respectively), but not in most of the symptom scales. The PhA is a useful tool for assessing the subjective health perception of cancer patients, especially with regard to psychological factors. While there is a statistically significant correlation, further research is required before confidently applying it in clinical practice. The current predictive value of this predictor for certain psychological aspects is limited, underscoring the need for additional research.

## 1. Introduction

The phase angle (PhA) is used to evaluate nutritional status and is an indicator of cellular health [1]. Clinically, the PhA reflects cell membrane function and body cell mass, which means that the higher the PhA, the better the cell function [2]. The principle behind the PhA concept relies on alterations in both resistance and reactance when alternating current flows through analyzed tissues. As the current traverses cellular membranes, a portion of it is stored within the resistive sections, resulting in a phase shift [2]. As a general rule, it is negatively correlated with the ratios of extra-to-intracellular water, age, and body mass index (BMI) but positively associated with physical activity [3,4]. 

The PhA’s impact has been evaluated in a wide range of malignancies and diseases such as sarcopenia [5,6,7], acute stroke [8], chronic obstructive pulmonary disease [8,9], liver cirrhosis [10], severe obesity [2], locomotive syndrome [11], cancer [12] and even in outpatient palliative cancer care settings [13]. In cancer patients, the PhA is considered a prognostic factor for survival [14,15], and it can predict postoperative infectious complications [16]. The PhA is frequently lower than normal in disease patients since processes such as inflammation, infection, or disease-specific events may impair the PhA [17]. Such alterations in the PhA can be measured through bioelectrical impedance analysis (BIA), a simple, fast, non-invasive and reproducible tool to assess body composition [18]. Furthermore, it can be performed at the bedside of critical patients to measure body composition [19]. The PhA has also been used as a nutritional status marker because the measured values reflect the amounts of various tissue components and hydration status [2]. The results of previous research suggest that there are some alterations in the electrical properties of tissues with malnutrition that can be detected by BIA, giving alterations in the PhA values. Currently, there are no cut-off points for malnutrition states but there are cut-off points for sarcopenia and frailty patterns in older adults, with a PhA value of ≤ 4.1 degrees being risky values [20].

In the assessment of sarcopenia and frailty in cancer patients, often due to nutritional problems, it is vital to record parameters related to these because, due to the side effects of treatments, many patients suffer from these conditions. The presence of sarcopenia in older adults has been related to increased functional impairment, disability, risk of falls and fractures, loss of independence, and death [21].

Another aspect to consider in cancer patients is their perception of their health. This is usually measured by different questionnaires such as the general health status indicator, which measures eight dimensions of health: physical function, physical role, bodily pain, general health, vitality, social function, emotional role and mental health (SF-36) in primary care [22,23] and in cancer [24,25]. The EORTC QLQ-C30 questionnaire encompasses a set of distinct components designed to evaluate the impact of cancer on individuals. It consists of five functional items, namely physical role, cognitive, emotional, and social functioning. In addition, it includes three symptom items focusing on fatigue, pain, and nausea–vomiting. Furthermore, there is a single item dedicated to assessing the global health status and overall quality of life [26,27,28,29]. The states of fatigue in different aspects of life (FACIT) is a specific instrument that objectively assesses health-related quality of life in patients with advanced or life-limiting chronic diseases [26,30]. Finally, the perception of general health (GHQ-12) is a screening instrument that aims to detect psychological morbidity and possible cases of psychiatric disorders in settings such as primary care [31,32,33]. The questionnaires chosen are all validated and have been used in previous research. They are also widely used by health professionals due to their reliability. Although there is a wide variety of questionnaires, these were chosen according to the criteria of mental health specialists. Some questionnaires are specific to the disease studied.

In previous research, it has been shown that the psychological state of cancer patients is significantly worsened compared to the healthy population. Psychological stressors for the majority of the population, such as COVID-19 [34,35,36], had no effect on cancer patients, possibly due to the previous psychological impairment of the patients [37].

A previous study by our research group has shown the relationship between the PhA and physical performance [38]. However, to our knowledge, the relationship between PhA and psychological aspects has not been discussed. To fill this gap, this study used the GHQ-12, FACIT, SF-36 and QLQ-C30 items to relate questionnaire results to the PhA values in cancer patients.

Regarding the use of PhA, a recent systematic review with meta-analysis explained that predicting survival in patients with advanced cancer remains a challenge but findings suggest that the PhA may be an important factor in this population [39].

Therefore, and considering the above, this article aims to: (1) evaluate the relationship between PhA and physiological state and (2) be able to use a predictive methodology for psychological state using the PhA as a measured value.

## 2. Methods

### 2.1. Study Design

The implementation of the program was overseen by the Primary Care Research Unit, which operates under the Bizkaia-Biocruces Bizkaia Research Institute and the University of Deusto. This initiative is executed through a network of health centers that are equipped with Bizi Orain exercise laboratories. These laboratories are seamlessly integrated into the public health system of the Basque Country, known as Osakidetza. The present study adopted a descriptive cross-sectional design and focused on a sample of individuals who have received a cancer diagnosis. Bizi Orain is an evidence-based exercise program that aligns with the guidelines established by the American College of Sports Medicine for cancer survivors [40]. The study lasted 12 months and was divided into two phases. During the first three months, participants were randomly assigned to a group that received both the Bizi Orain exercise program and a behavioral intervention to promote healthy habits, or to a group that received only the healthy habits prescription. After three months, participants in the second group started the exercise program. During the last six months of the study, participants continued with their daily activities and were evaluated at 6 and 12 months before the end of their participation in the study. During this time, PhA measurements were obtained and used for further data analysis. 

Each study participant was informed of all study procedures, signed the consent form about the tests performed, and completed all study procedures. 

### 2.2. Participants

Anyone with a cancer diagnosis currently receiving treatment or diagnosed less than two years earlier was eligible to participate. The participants included in this study were referred by their respective oncologists or hematologists at Cruces, Basurto, and Galdakao University Hospitals, located in Bizkaia, Basque Country, Spain. These referrals were made as part of the main project, which established the study’s selection and exclusion criteria in detail [41]. 

To ensure ethical compliance, this study adheres to the principles outlined in the Declaration of Helsinki and its subsequent revisions, as well as good clinical practice. The Ethics Committee of the Basque Country granted approval for this study to be conducted in the health centers, guaranteeing compliance with all relevant regulations (Code: PI2019016).

Regarding data confidentiality, only the designated study researchers had access to patient data, which was provided voluntarily by the participants. The handling of this data adhered to the requirements outlined in the Organic Act 15/1999 of December 2013, which concerns the protection of personal data, as well as its 2011 revision.

It is important to note that this project is part of a broader initiative focusing on recovery, stress balance, and injury in sports, which received approval from the Institutional Review Board. All participants involved in the study provided informed consent prior to their involvement.

### 2.3. Measurements

During the period spanning July 2019 to July 2022, patients underwent physical and psychological evaluations at the University of Deusto (Spain). The assessments were conducted at the time of study enrollment and subsequently at 3-, 6-, and 12-month intervals. Consistent with maintaining standardization, these evaluations were conducted within the same time frame each day, specifically from 9:00 to 14:00. Furthermore, the evaluations took place under similar environmental conditions, ensuring that the temperature remained at approximately ±21 °C, the relative humidity was maintained between 50 and 55%, and the barometric pressure hovered around ±720 mmHg.

The patients’ height was measured utilizing a wall stadiometer (Seca, Hamburg, Germany). In addition, body composition measurements were obtained using an Inbody 770 bioimpedance analyzer (Inbody, Seoul, Korea). These measurement procedures strictly adhered to the established protocol to ensure accuracy and consistency [42]. The use of the Inbody 770 device as a meter is validated against the gold standard DEXA with 98% correlation [43]. The measurements were taken standing up because research has shown that position from which the PhA is taken interferes with some of the values, so the way in which the bioimpedance was measured should always be known [44]. The PhA is compounded by two elements: the reactance (X) and the resistance (R). It should be noted that this measurement is a direct assessment of the cell membrane, and not a calculation based on indirect equations. The formula for the phase angle is:

φ = arctg X/R

A series of questionnaires with sound psychometric properties were used to assess general health, cancer-specific quality of life, and cancer-related fatigue. These questionnaires were those used in practice by the clinicians in the hospitals involved in the study. The Medical Outcomes Study 36-Item Short-Form Health Survey (SF-36) is used to assess general health-related quality of life status [23,41], and cancer-specific quality of life is assessed by the Core Quality of Life Questionnaire (QLQ-C-30). The SF-36 consists of eight scaled scores obtained through the weighted sums of the questions in each section. Each scale is directly transformed into a 0–100 scale, assuming that each question carries equal weight. The lower the score, the higher the impediment, the higher the score, the lower the disability [29,41]. The General Health Questionnaire (GHQ-12) assesses psychological morbidity and possible psychiatric disorders [32,41]. Items on the GHQ-12 are rated on a 4-point scale using a timeframe of “the last two weeks.” There are three ways of scoring the GHQ-12: the bimodal GHQ scoring method (0-0-1-1), recommended by the test authors for use in clinical settings; the Likert scoring method (0-1-2-3), which is commonly used in research; and the C-GHQ scoring method where positively phrased items are scored (0-0-1-1) and negatively phrased items (0-1-1-1) [45]. The more severe the problem, the higher the score (with a maximum of 9 for each of the subscales). Finally, cancer-related fatigue is assessed using the Functional Assessment of Chronic Illness Therapy–Fatigue (FACIT-Fatigue) scale [28,41]. The FACIT-Fatigue Scale is a short, 13-item, easy-to-administer tool that measures an individual’s level of fatigue during their usual daily activities over the past week. The level of fatigue is measured on a four-point Likert scale (4 = not at all fatigued to 0 = very much fatigued), lower test scores indicate greater fatigue [46].

### 2.4. Statistical Analysis

Mixed linear regression models were employed to examine the association between the PhA and the psychological tests (SAS: PROC MIXED), which accommodate the inherent correlations observed in repeated measurements within each patient. Adjustment variables such as age, sex, cancer type, stage, treatments, smoking status, and comorbidities were incorporated into the analysis. Importantly, none of these variables exhibited any missing values.

A stepwise backward strategy was employed to identify the most appropriate model, guided by likelihood ratio tests. The selection process involved iteratively removing variables that did not significantly contribute to the model’s explanatory power, with a significance criterion set at *p* < 0.05. The final model was determined by following this rigorous approach, ensuring a robust and reliable analysis. We reported the proportion of variance explained by the fixed variables in the models using the marginal R square obtained by the Nakagawa method [47]. We also performed correlation tests between the PhA and the psychological variables. All analyses were performed with SAS 9.4 and R.

## 3. Results

Of the 311 patients included in the study, 309 had at least one PhA measurement, 239 had at least two measurements, 205 had at least three measurements, and 121 had four measurements. Therefore, the total data are 874 PhA measurements; each accepted measurement of PhA had to go with the corresponding results of the questionnaires, thus providing the data of both PhA and the psychological situation at each moment, Within the mixed statistical model, the special feature of these measurements is taken into account. Table 1 shows the sample characteristics according to the main descriptive variables. The results of the questionnaires are presented in Table A2.

With regard to the results shown in Table 2, we appreciate the significant relationship that exists between GHQ-12 and FACIT values and PhA. It is known that the lower the score in the GHQ-12-questionnaire values, the better the general state of health of the patient and the higher the PhA, all with a high significance. Furthermore, observing the FACIT questionnaire, the higher the score, the lower the fatigue perceived by the questionnaire. A relationship was also observed between the FACIT questionnaire score and the PhA value.

Continuing with the results obtained, a high significance can also be seen between the SF-36 questionnaire and the PhA. Specifically, in the subclassification made by the questionnaire, a high relationship can be seen, except in the subclassification of the emotional role and pain index, where a relationship with the PhA cannot be seen. This questionnaire is interpreted as meaning that the lower the score, the greater the patient’s limitation in each of the subclassifications.

The QLQ-C30 questionnaire interprets higher scale scores as higher response levels. It is divided into two main scales, the functioning scale and the symptom scale. In the functioning scale, we can appreciate the relationship with the PhA except in the emotional and cognitive functioning parameters, which are not related to the PhA. In the symptom scale, only the parameters of fatigue, nausea and vomiting, and loss of appetite are related to PhA, with no clear relationship in the rest of the subclassifications. Further analysis and supplementary results can be found in Table A1.

Finally, we can see how the pain scale is measured using different questionnaires, SF-36 and QLQ-C30, and the relationship between this parameter and PhA was not significant in either case.

Further statistical analyses not relevant to the research have been added in Table A1.

## 4. Discussion

This study analyzed the possible associations between the PhA and the cancer patients’ subjective perception of several health parameters. Among the most relevant results, we found that while the relationship with PhA was high in the GHQ-12, FACIT, and SF-36 questionnaires, with higher PhA being associated with better results, a relationship with PhA was found in the functioning scale assessment items of the QLQ-C30 but not in most of its symptom scales.

Regarding the patient’s subjective assessment, the GHQ-12 questionnaire measures possible affective disorders in different populations [31]. Thus, as a valid tool, finding a high relationship with the PhA could help us predict affective disorders with other tools, such as the PhA. The results obtained in our research show how the two values, both the GHQ-12 score and the PhA, are closely correlated, with higher GHQ-12 values leading to higher PhA values.

In the same way that previous research has shown a relationship between PhA and better physical profiles [38], it is not uncommon to observe the same trend of higher PhA values with better psychological profiles. The relationship that PhA has with physical and psychological aspects could lead to the hypothesis that the better the physical health, the better the psychological health [48] and, therefore, the higher the PhA. Another possible hypothesis for this relationship between GHQ-12 values and PhA could be cellular integrity since patients with a better psychological profile could be those with more favorable cellular integrity [49].

Cancer-related fatigue is a common side effect of cancer treatment, so the fatigue questionnaire (FACIT) has long been used to assess and monitor this side effect [50], especially as a part of physical exercise programs. Our research has shown a close relationship between the values of this questionnaire and the PhA values measured, showing that higher PhA levels would result in lower fatigue as measured by the questionnaire. This result was discussed in previous research where a relationship between fatigue and PhA was observed, especially in cancer patients with PhA <4, but when adjusted for hydration parameters (intracellular and extracellular water), the association was reduced [51]. The relationship between these two parameters, fatigue and PhA, has an important relevance given that PhA values can be related to the fatigue questionnaire (FACIT) value, knowing that, in our study, for each one-grade increase in PhA, an improvement of 3.2 (1.99; 4.41) points in FACIT scores could be seen with a high relationship. This relationship between fatigue and PhA could be explained by the fact that PhA is an indicator of physical health in cancer patients [38], so lower PhA values indicate poorer physical condition, which could easily be associated with higher fatigue.

Looking at the SF-36 questionnaire results, which measured health-related quality of life, we observed a high relationship with the values obtained in PhA values. It is known that on most occasions, a better quality of life is preceded by lifestyle changes, and this positively affects PhA. It has been seen that improving nutritional status by improving diet [52], practicing planned physical exercise or improving body composition [53,54] can lead to an improved quality of life and PHA. Therefore, improvement in any aspect that aids quality of life positively affects both SF-36 and PhA values.

The subsections measured by this SF-36 questionnaire are highly related to PhA with values of *p* < 0.05, except for the parameters of emotional role and pain, which have not been found to have a significant relationship. Emotional role measures the degree to which emotional problems interfere with work or other daily activities, so it can be extrapolated that there is no relationship between PhA and this measure, possibly because, in many cases, psychological problems do not affect their daily activities, although a large proportion report episodes of depression and anxiety [55]. These results may also be biased by possible psychiatric medication to control these depression and anxiety problems because at least 25–30% of cancer patients and an even higher percentage of those with advanced disease meet the criteria for a psychiatric diagnosis, including depression, anxiety, stress-related syndromes, adjustment disorders, sleep disorders, and delirium [56]. Previous studies showed that emotional role and one’s physical state negatively influenced each other, while fatigue and pain positively influenced each other. These results suggest that mind and body interact in a direct way [57], with differences in the results in our research.

The results obtained in the QLQ-C30, specifically those corresponding to the measurement of functioning scales, show how emotional functioning is unrelated to PhA. It should be noted that this same section was assessed in the SF-36 questionnaire, giving similar data regarding the non-significance of the relationship with PhA, confirming the non-significance of the relationship with PhA with two different tools. This could be because, as we have previously stated, the better or worse value of PhA is independent of the problems that the illness may cause in work or daily activities. Nutrition plays a fundamental role in the quality of life [52] and in the possibility of modifying PhA values. Therefore, a parameter related to nutrition could be the item that speaks of appetite loss measured with the QLQ-C30, which has a significant relationship with PhA. Thus, patients with low PhA have a significant loss of appetite.

The PhA’s relationship with pain is not significant for the results in the QLQ-C30 and the SF-36 questionnaires. The same parameter, measured with two different instruments, provided us with the important information that it does not have a relationship with the PhA. 

Due to the novelty of the analyzed parameters and the relative association between the parameters, more and better research is needed to be able to apply this knowledge in daily practice although this research lays the foundation for further research regarding PhA in the management of cancer patients.

It is important to note that bioimpedance measurements obtained from single and multifrequency devices should not be treated as interchangeable. When evaluating highly active populations, there was a notable lack of consensus between devices when determining individual values such as R, Xc, Z, and PhA [58]. This discrepancy could be attributed to various methodological and biological factors. Consequently, if this methodology is to be applied in clinical practice, it is strongly advised to consistently use the same device, conduct measurements under as similar conditions as possible, and refrain from comparing results with data obtained from different devices or methodologies.

### 4.1. Limitations

There is evidence that PhA measurements can vary according to the measuring device and body position at the time of assessment (standing vs. supine) [44]. During this research, all measurements were made standing to avoid variance.

The sample was taken from a larger project that included cancer patients of all types and stages, so in order to have a good sample it was decided to include all patients knowing the limitation of the heterogeneity of the sample and its possible relationship to the results.

Mixed linear regression models may have limitations in interpretation and more research is needed to apply these regressions to everyday practice to obtain the desired uses.

### 4.2. Practical Applications

This research opens up a new possibility of having the tools to monitor fatigue in patients whose PhA drops. That is, FACIT questionnaires may be used to assess patient fatigue. 

The relationship between most of the items in the questionnaire and the PhA opens up the possibility of carrying out a quick screening, knowing which patients would need more research into their subjective perception of health.

## 5. Conclusions

This study analyzed the possible associations between the PhA and cancer patients’ subjective perception of different health parameters. It could be seen that while in the GHQ-12, FACIT and SF-36 questionnaires, there was a correlation with PhA, with higher PhA being seen with better results in the questionnaires; in other questionnaires, such as the QLQ-C30, a relationship with PhA was seen in the functioning scale assessment items but not in the majority of the symptom scales. Therefore, the PhA is a good tool to assess the subjective health perception of a cancer patient and due to the regressions can make predictions of outcomes except for some items where the association is not significant. It should be noted that the correlation is low, despite being statistically significant, so the actual predictive value is limited.

## Figures and Tables

**Table 1:** Characteristics of the sample. The data is shown as mean ± standard deviation (SD) or percentage (%).

Variable	n (%)	Mean ± SD
AGE (years)	311 (100)	55.5 ± 10.9
SEX		-
FEMALE	225 (72.3)	
MALE	86 (27.65)	
HEIGHT (cm)		164.7 ± 8.7
WEIGHT (kg)		70.9 ± 14.5
BMI (kg/m^2^)		26.1 ± 4.9
PhA (°)		4.9 ± 0.7
VO_2_peak (mL/kg/min)		15.3 ± 4
CHEMOTHERAPY	274 (88.1)	
RADIOTHERAPY	92 (29.6)	
BONE METASTASES	50 (16.1)	
SURGERY	164 (52.9)	
CANCER STAGE		
I	48 (15.4)	
II	80 (25.7)	
III	62 (19.9)	
IV	121 (38.9)	
CANCER TYPE		
Breast	132 (42.4)	
Colorectal	24 (7.7)	
Hematologic	69 (22.2)	
Ovary	14 (4.5)	
Pancreas	17 (5.5)	
Lung	19 (6.1)	
Other	36 (11.6)	
SMOKING		
Never	169 (54.3)	
Ex-smoker	94 (30.2)	
Smoker	48 (15.4)	
COMORBIDITIES		
Diabetes	27 (8.7)	
COPD	16 (5.1)	
Heart failure	8 (2.6)	
Hypertension	68 (21.9)	
Hyperlipidemia	70 (22.5)	

BMI: body mass index; PhA: phase angle; VO2peak: peak consume of VO_2_; COPD: chronic obstructive pulmonary disease.

**Table 2:** Relationship between PhA and questionnaires.

Questionnaire	Mixed Linear Regression Estimate (95% CI) Size Effect	Mixed Linear Regression *p*-Value	R^2^	Pearson Coefficient Correlation (95%CI)	Correlation *p*-Value
GHQ-12	−0.72 (−1.16; −0.27)	0.002 *	0.05 (0.01; 0.12)	−0.05 (−0.12; 0.02)	0.132
FACIT	3.2 (1.99; 4.41)	<0.001 *	0.08 (0.04; 0.16)	0.16 (0.09; 0.22)	<0.001 *
SF-36					
Physical functioning	7.99 (5.54; 10.44)	<0.001 *	0.14 (0.09; 0.21)	0.23 (0.17; 0.3)	<0.001 *
Social functioning	9.13 (5.67; 12.54)	<0.001 *	0.06 (0.03; 0.12)	0.11 (0.04; 0.17)	<0.001 *
Physical role	13.03 (7.4; 18.59)	<0.001 *	0.08 (0.03; 0.14)	0.09 (0.02; 0.15)	0.009 *
Emotional role	1.88 (−3.91; 7.62)	0.525	0.04 (0.02; 0.11)	−0.01 (−0.07; 0.06)	0.829
Mental health	4.34 (1.76; 6.9)	<0.001 *	0.10 (0.04; 0.20)	0.11 (0.05; 0.18)	<0.001 *
Energy/vitality	8.2 (5.38; 11)	<0.001 *	0.12 (0.07; 0.22)	0.18 (0.12; 0.25)	<0.001 *
Pain index	1.75 (−1.55; 5.03)	0.296	0.08 (0.04; 0.16)	0.05 (−0.02; 0.11)	0.176
General health perception	5.17 (2.78; 7.55)	<0.001 *	0.07 (0.04; 0.16)	0.12 (0.05; 0.18)	<0.001 *
Standardized physical comp.	3.02 (1.79; 4.22)	<0.001 *	0.10 (0.08; 0.20)	0.16 (0.1; 0.23)	<0.001 *
Standardized mental comp.	2.31 (0.68; 3.92)	0.006 *	0.06 (0.02; 0.14)	0.05 (−0.01; 0.12)	0.120
QLQ-C30					
*Functioning scales*					
Global health status	5.63 (2.91; 8.33)	<0.001 *	0.06 (0.02; 0.11)	0.1 (0.04; 0.17)	0.003 *
Physical functioning	5.22 (3.34; 7.09)	<0.001 *	0.11 (0.07; 0.18)	0.22 (0.15; 0.28)	<0.001 *
Role functioning	7.86 (4.64; 11.05)	<0.001 *	0.11 (0.08; 0.20)	0.14 (0.07; 0.2)	<0.001 *
Emotional functioning	2.06 (−0.73; 4.84)	0.148	0.08 (0.03; 0.15)	0.02 (−0.05; 0.09)	0.538
Cognitive functioning	1.62 (−1.12; 4.36)	0.248	0.05 (0.02; 0.13)	0.07 (0.01; 0.14)	0.030 *
Social functioning	8.23 (4.66; 11.75)	<0.001 *	0.10 (0.05; 0.20)	0.08 (0.01; 0.14)	0.021 *
*Symptom scale/items*					
Fatigue	−6.37 (−9.34; −3.36)	<0.001 *	0.09 (0.04; 0.18)	−0.12 (−0.19; −0.06)	<0.001 *
Nausea and vomiting	−2.15 (−3.81; −0.49)	0.012 *	0.02 (0.01; 0.06)	−0.06 (−0.13; 0)	0.057
Pain	−1.07 (−4.19; 2.06)	0.504	0.07 (0.03; 0.14)	−0.03 (−0.1; 0.03)	0.310
Dyspnoea	−1.58 (−4.8; 1.69)	0.341	0.08 (0.05; 0.18)	−0.05 (−0.11; 0.02)	0.152
Insomnia	0.58 (−3.56; 4.73)	0.786	0.03 (0.01; 0.10)	−0.01 (−0.07; 0.06)	0.819
Appetite loss	−3.73 (−6.38; −1.08)	0.006 *	0.07 (0.04; 0.18)	−0.1 (−0.16; −0.03)	0.004 *
Constipation	−2.79 (−6; 0.41)	0.089	0.05 (0.02; 0.13)	−0.05 (−0.12; 0.02)	0.142
Diarrhoea	−2.62 (−5.34; 0.1)	0.061	0.09 (0.05; 0.18)	−0.02 (−0.08; 0.05)	0.606
Financial difficulties	0.44 (−2.99; 3.87)	0.803	0.06 (0.03; 0.13)	0.06 (−0.01; 0.12)	0.101

* statistically significant.

## Data Availability

Regarding data confidentiality, only the study researchers have access to the data of individuals who agreed to participate in the study, in compliance with the Organic Act 15/1999 of December 2013 on the protection of personal data and its 2011 revision.

## Appendix A

**Table A1:** Supplementary analysis.

Questionnaire				Estimate	95%CI	*p*-Value
**SF36**	**Physical Functioning**					
		Intercept		47.26	(33.64; 61.08)	<0.001
		PhA		7.08	(4.62; 9.51)	<0.001
		Sex				0.079
			Male	Ref		
			Female	−4.07	(−8.54; 0.41)	
		Smoking				0.018
			Never	Ref		
			Ex-smoker	−6.2	(−10.48; −1.92)	
			Smoker	−3.13	(−8.55; 2.29)	
		Bone metastases		−6.27	(−11.43; −1.09)	0.019
		Hypertension		−7.85	(−13; −2.71)	0.003
		Hyperlipidemia		−4.53	(−9.57; 0.53)	0.083
	**Physical Role**					
		Intercept		−2.51	(−37.87; 33.59)	0.890
		PhA		10.89	(5.2; 16.45)	<0.001
		Age				<0.001
			<45	Ref		
			<60	−2.89	(−18.21; 12.37)	
			<70	20.85	(2.97; 38.68)	
			70+	42.33	(0.64; 84.03)	
		Sex				0.034
			Male	Ref		
			Female	−9.66	(−18.52; −0.85)	
		Hypertension		−16.73	(−26.2; −7.29)	<0.001
	**Pain Index**					
		Intercept		55.64	(33.86; 77.81)	<0.001
		PhA		3.25	(−0.17; 6.61)	0.062
		Age				0.01
			<45	Ref		
			<60	−2.87	(−12.7; 6.92)	
			<70	7.21	(−4.26; 18.67)	
			70+	21.13	(−5.47; 47.84)	
		Sex				<0.001
			Male	Ref		
			Female	−10.55	(−16.21; −4.91)	
		Smoking				0.047
			Never	Ref		
			Ex-smoker	−6.38	(−11.63; −1.11)	
			Smoker	0.26	(−6.43; 6.98)	
		Hypertension		−7.47	(−13.53; −1.45)	0.017
	**General Health**					
		Intercept		34.02	(21.88; 46.22)	<0.001
		PhA		4.25	(1.98; 6.5)	<0.001
		Stage				0.001
			I	Ref		
			II	0.93	(−5.16; 7.01)	
			III	−0.83	(−7.31; 5.66)	
			IV	−7.64	(−13.38; −1.89)	
		Diabetes		9.08	(2.2; 15.96)	0.011
		COPD		−7.39	(−16.03; 1.24)	0.097
	**Vitality**					
		Intercept		14.77	(−2.85; 32.64)	0.106
		PhA		7.74	(5; 10.43)	<0.001
		Age				0.003
			<45	Ref		
			<60	3.92	(−4.27; 12.11)	
			<70	13.14	(3.68; 22.61)	
			70+	20.66	(−0.99; 42.39)	
		Sex				0.027
			Male	Ref		
			Female	−5.34	(−10.02; −0.69)	
		Smoking				0.023
			Never	Ref		
			Ex-smoker	−5.64	(−10.04; −1.23)	
			Smoker	1.21	(−4.56; 6.99)	
		COPD		−10.4	(−19.62; −1.2)	0.029
	**Social Functioning**					
		Intercept		21.33	(1.58; 41.36)	0.035
		PhA		8.96	(5.58; 12.27)	<0.001
		Age				<0.001
			<45	Ref		
			<60	4.86	(−5.34; 15.05)	
			<70	17.38	(5.67; 29.07)	
			70+	26.31	(−1.01; 53.65)	
		Hyperlipidemia		−5.96	(−12.03; 0.11)	0.057
	**Emotional Role**					
		Intercept		62.26	(26.66; 98.22)	<0.001
		PhA		0.86	(−4.75; 6.42)	0.764
		Age				0.024
			<45	Ref		
			<60	9.7	(−6.23; 25.59)	
			<70	24.22	(5.89; 42.48)	
			70+	14.73	(−27.63; 57.06)	
		Sex				0.062
			Male	Ref		
			Female	−8.76	(−17.87; 0.31)	
		COPD		−19.64	(−37.41; −1.93)	0.032
	**Mental Health**					
		Intercept		43.61	(26.19; 61.32)	<0.001
		PhA		4.03	(1.5; 6.52)	0.002
		Age				0.012
			<45	Ref		
			<60	2.42	(−5.6; 10.42)	
			<70	10.82	(1.65; 19.98)	
			70+	10.99	(−10.28; 32.26)	
		Sex				0.007
			Male	Ref		
			Female	−6.62	(−11.34; −1.94)	
		Stage				0.032
			I	Ref		
			II	9.19	(3.02; 15.35)	
			III	4.12	(−2.48; 10.72)	
			IV	5.65	(−0.27; 11.56)	
		COPD		−9.76	(−18.64; −0.91)	0.034
	**Standarized Physical Component**					
		Intercept		28.31	(21.85; 34.9)	<0.001
		PhA		3.17	(2.07; 4.25)	<0.001
		Age				0.054
			<45	Ref		
			<60	−1.35	(−4.71; 2.01)	
			<70	1.51	(−2.36; 5.38)	
			70+	4.11	(−5.01; 13.23)	
		Smoking				0.068
			Never	Ref		
			Ex-smoker	−1.92	(−3.73; −0.11)	
			Smoker	0.53	(−1.76; 2.82)	
		Bone metastases		−2.1	(−4.29; 0.09)	0.064
		Hypertension		−3.96	(−6.02; −1.91)	<0.001
	**Standarized Mental Component**					
		Intercept		28.05	(18.94; 37.21)	<0.001
		PhA		2.51	(0.99; 4.02)	0.001
		Age				<0.001
			<45	Ref		
			<60	3.85	(−1.06; 8.76)	
			<70	9.77	(4.21; 15.34)	
			70+	8.98	(−4.24; 22.22)	
		Diabetes		4.19	(−0.15; 8.52)	0.061
		COPD		−5.69	(−11.14; −0.26)	0.043
**EORT QLQ-30**						
	**Global Health Status/QoL**					
		Intercept		32.54	(17.73; 47.4)	<0.001
		PhA		5.56	(3.06; 8.05)	<0.001
		Age				<0.001
			<45	Ref		
			<60	1.4	(−6.18; 8.97)	
			<70	12.13	(3.57; 20.7)	
			70+	8.98	(−11.1; 29.1)	
		COPD		−8.83	(−17.24; −0.43)	0.042
	**Physical functioning**					
		Intercept		67.11	(57.64; 76.68)	<0.001
		PhA		4.72	(3; 6.43)	<0.001
		Sex				0.019
			Male	Ref		
			Female	−3.8	(−6.95; −0.66)	
		Bone metastases		−4.76	(−8.44; −1.07)	0.013
		COPD		−6.53	(−12.77; −0.3)	0.042
		Hyperlipidemia		−4.46	(−7.72; −1.2)	0.008
	**Role functioning**					
		Intercept		50.65	(29.75; 72.08)	<0.001
		PhA		6.7	(3.55; 9.78)	<0.001
		Age				<0.001
			<45	Ref		
			<60	−0.18	(−8.62; 8.19)	
			<70	13.89	(4.05; 23.67)	
			70+	31.91	(9.15; 54.64)	
		Sex				0.044
			Male	Ref		
			Female	−6.19	(−12.09; −0.36)	
		Cancer type				0.034
			Breast	Ref		
			Colorectal	−4.95	(−12.86; 2.97)	
			Hematologic	−3.22	(−9; 2.54)	
			Ovary	−4.65	(−13.74; 4.4)	
			Pancreas	−10.53	(−20.7; −0.5)	
			Lung	9.66	(0.35; 18.85)	
			Other	−4.15	(−11.74; 3.45)	
		Bone metastases		−5.84	(−11.33; −0.3)	0.043
		COPD		−14.17	(−23.88; −4.53)	0.005
		Hypertension		−4.61	(−9.82; 0.55)	0.09
	**Emotional functioning**					
		Intercept		67.18	(49.51; 84.98)	<0.001
		PhA		1.86	(−0.87; 4.57)	0.181
		Age				0.002
			<45	Ref		
			<60	4.75	(−3.64; 13.14)	
			<70	14.21	(4.65; 23.76)	
			70+	18.54	(−3.69; 40.77)	
		Sex				<0.001
			Male	Ref		
			Female	−8.33	(−13.1; −3.57)	
	**Cognitive functioning**					
		Intercept		88.44	(74.3; 102.62)	<0.001
		PhA		0.52	(−2.06; 3.09)	0.693
		Sex				<0.001
			Male	Ref		
			Female	−9.69	(−14.57; −4.81)	
		COPD		−8.29	(−17.94; 1.37)	0.094
	**Social functioning**					
		Intercept		21.45	(2.12; 40.98)	0.032
		PhA		7.16	(3.88; 10.41)	<0.001
		Age				<0.001
			<45	Ref		
			<60	16.87	(6.88; 26.85)	
			<70	31.68	(20.4; 42.94)	
			70+	26.44	(−0.49; 53.39)	
		Bone metastases		−7.87	(−14.43; −1.31)	0.02
		Diabetes		7.86	(−0.99; 16.69)	0.085
	**Fatigue**					
		Intercept		55.77	(36.81; 74.41)	<0.001
		PhA		−5.65	(−8.5; −2.75)	<0.001
		Age				0.048
			<45	Ref		
			<60	−1.36	(−10.02; 7.32)	
			<70	−9.29	(−19.28; 0.68)	
			70+	−7.64	(−30.26; 15.05)	
		Sex				0.015
			Male	Ref		
			Female	6.12	(1.27; 11)	
		Smoking				0.039
			Never	Ref		
			Ex-smoker	5.89	(1.3; 10.48)	
			Smoker	0.6	(−5.45; 6.63)	
		COPD		13.16	(3.58; 22.8)	0.008
	**Nausea and vomiting**					
		Intercept		12.1	(3.79; 20.36)	0.005
		PhA		−1.61	(−3.18; −0.02)	0.047
		Stage				0.041
			I	Ref		
			II	−0.16	(−3.78; 3.46)	
			III	4.24	(0.4; 8.07)	
			IV	1.99	(−1.43; 5.41)	
	**Pain**					
		Intercept		20.28	(0.93; 39.57)	0.042
		PhA		−1	(−4.01; 2.01)	0.516
		Age				0.021
			<45	Ref		
			<60	4.69	(−4.05; 13.43)	
			<70	−2.54	(−12.76; 7.65)	
			70+	−18.71	(−42.47; 4.98)	
		Sex				<0.001
			Male	Ref		
			Female	10.98	(5.96; 16.01)	
		COPD		9.12	(−0.56; 18.84)	0.069
		Hypertension		5.23	(−0.16; 10.63)	0.061
	**Dyspnoea**					
		Intercept		10.32	(−5.88; 26.26)	0.21
		PhA		−1.32	(−4.19; 1.6)	0.373
		Sex				<0.001
			Male	Ref		
			Female	9.61	(4.66; 14.56)	
		Smoking				0.008
			Never	Ref		
			Ex-smoker	7.28	(2.59; 11.95)	
			Smoker	4.71	(−1.38; 10.78)	
		COPD		15.08	(5.32; 24.85)	0.003
		Hypertension		8.54	(3.35; 13.76)	0.002
	**Insomnia**					
		Intercept		18.28	(−2.5; 39.05)	0.085
		PhA		1.2	(−2.63; 5.02)	0.54
		Sex				<0.001
			Male	Ref		
			Female	13.09	(6.44; 19.73)	
	**Appetite loss**					
		Intercept		12.55	(−4.63; 29.61)	0.157
		PhA		−2.39	(−5.15; 0.42)	0.098
		Sex				0.001
			Male	Ref		
			Female	9.39	(3.83; 14.94)	
		Cancer type				0.002
			Breast	Ref		
			Colorectal	10.61	(2.67; 18.52)	
			Hematologic	0.67	(−4.98; 6.35)	
			Ovary	−5.77	(−14.99; 3.47)	
			Pancreas	18.05	(8.1; 28.02)	
			Lung	6.64	(−2.17; 15.46)	
			Other	5.87	(−1.7; 13.47)	
		Stage				0.042
			I	Ref		
			II	−0.73	(−6.88; 5.39)	
			III	7.62	(0.74; 14.42)	
			IV	2.29	(−4.01; 8.53)	
	**Constipation**					
		Intercept		30.48	(14.37; 46.39)	<0.001
		PhA		−3.25	(−6.38; −0.08)	0.046
		Smoking				0.042
			Never	Ref		
			Ex-smoker	6.96	(1.58; 12.35)	
			Smoker	1.33	(−5.44; 8.09)	
		Cancer type				0.023
			Breast	Ref		
			Colorectal	−1.46	(−10.42; 7.52)	
			Hematologic	−5.04	(−11.14; 1.04)	
			Ovary	17.05	(5.92; 28.2)	
			Pancreas	3.07	(−8.14; 14.24)	
			Lung	3.69	(−6.38; 13.78)	
			Other	1.52	(−6.54; 9.53)	
	**Diarrhoea**					
		Intercept		26.21	(10.07; 42.18)	0.002
		PhA		−1.7	(−4.21; 0.87)	0.201
		Age				0.006
			<45	Ref		
			<60	−11	(−18.26; −3.86)	
			<70	−14.35	(−22.69; −6.15)	
			70+	−24.68	(−44.46; −5.07)	
		Smoking				0.038
			Never	Ref		
			Ex-smoker	4.93	(0.94; 8.93)	
			Smoker	−0.67	(−5.61; 4.31)	
		Cancer type				0.003
			Breast	Ref		
			Colorectal	9.92	(3.19; 16.44)	
			Hematologic	−3.35	(−8.9; 2.13)	
			Ovary	−4.7	(−12.75; 3.36)	
			Pancreas	7.51	(−1.11; 16.09)	
			Lung	−4.55	(−12.06; 3.01)	
			Other	0.5	(−5.65; 6.65)	
		Surgery		−4.47	(−8.64; −0.3)	0.043
		Chemotherapy		5.11	(−0.35; 10.61)	0.077
		Diabetes		9.8	(3.31; 16.51)	0.005
	**Financial difficulties**					
		Intercept		18.26	(−1.62; 38.09)	0.074
		PhA		0.5	(−2.71; 3.71)	0.763
		Age				0.002
			<45	Ref		
			<60	−3.48	(−14.73; 7.79)	
			<70	−16.66	(−29.39; −3.91)	
			70+	−20.17	(−49.84; 9.52)	
		Radiotherapy		−5.25	(−11.15; 0.66)	0.085
		COPD		13.4	(0.97; 25.84)	0.037
**Golberg [GHQ-12]**						
		Intercept		8.14	(5.4; 10.86)	<0.001
		PhA		−0.62	(−1.06; −0.18)	0.006
		Age				<0.001
			<45	Ref		
			<60	−1	(−2.37; 0.37)	
			<70	−2.52	(−4.06; −0.98)	
			70+	−3.67	(−7.32; −0.03)	
		Stage				0.062
			I	Ref		
			II	−1.42	(−2.47; −0.36)	
			III	−0.79	(−1.91; 0.33)	
			IV	−1.1	(−2.1; −0.11)	
**FACIT**						
		Intercept		26.96	(19; 35.01)	<0.001
		PhA		2.65	(1.41; 3.88)	<0.001
		Age				0.04
			<45	Ref		
			<60	−0.03	(−3.77; 3.7)	
			<70	3.5	(−0.78; 7.77)	
			70+	3.42	(−6.46; 13.32)	
		Sex				0.04
			Male	Ref		
			Female	−2.26	(−4.39; −0.14)	
		Bone metastases		−2.54	(−4.98; −0.1)	0.044
		COPD		−5.81	(−9.96; −1.67)	0.007

**Table A2:** General questionnaire original data.

Questionnaire	Mean (SD)
GHQ-12	3.36 (3.63)
FACIT	37.33 (9.53)
SF-36	
Physical functioning	70.92 (20.91)
Social functioning	66.68 (27.58)
Physical role	70.92 (20.91)
Emotional role	67.85 (43.61)
Mental health	66.1 (19.69)
Energy/vitality	50.13 (21.13)
Pain index	58.47 (26.24)
General health perception	51.19 (17.77)
Standardized physical comp.	40.32 (8.28)
Standardized mental comp.	44.21 (12.38)
QLQ-C30	
*Functioning scales*	
Global health status	60.77 (19.62)
Physical functioning	84.69 (14.72)
Role functioning	74.65 (25.57)
Emotional functioning	75.54 (20.18)
Cognitive functioning	82.85 (22.33)
Social functioning	70.98 (27.77)
*Symptom scale/items*	
Fatigue	33.64 (22.41)
Nausea and vomiting	7.2 (15.87)
Pain	27.33 (22.79)
Dyspnoea	15.86 (23.34)
Insomnia	35.6 (31.42)
Appetite loss	13.59 (22.68)
Constipation	18.23 (27.3)
Diarrhoea	12.19 (23.24)
Financial difficulties	13.03 (26.75)
